# Development and Internal Validation of a Nomogram to Predict Mortality During the ICU Stay of Thoracic Fracture Patients Without Neurological Compromise: An Analysis of the MIMIC-III Clinical Database

**DOI:** 10.3389/fpubh.2021.818439

**Published:** 2021-12-22

**Authors:** Haosheng Wang, Yangyang Ou, Tingting Fan, Jianwu Zhao, Mingyang Kang, Rongpeng Dong, Yang Qu

**Affiliations:** ^1^Department of Orthopedics, Second Hospital of Jilin University, Changchun, China; ^2^Department of Endocrinology, Baoji City Hospital of Traditional Chinese Medicine, Baoji, China

**Keywords:** intensive care units, nomogram, spinal fractures, prediction model, mortality

## Abstract

**Background:** This study aimed to develop and validate a nomogram for predicting mortality in patients with thoracic fractures without neurological compromise and hospitalized in the intensive care unit.

**Methods:** A total of 298 patients from the Medical Information Mart for Intensive Care III (MIMIC-III) database were included in the study, and 35 clinical indicators were collected within 24 h of patient admission. Risk factors were identified using the least absolute shrinkage and selection operator (LASSO) regression. A multivariate logistic regression model was established, and a nomogram was constructed. Internal validation was performed by the 1,000 bootstrap samples; a receiver operating curve (ROC) was plotted, and the area under the curve (AUC), sensitivity, and specificity were calculated. In addition, the calibration of our model was evaluated by the calibration curve and Hosmer-Lemeshow goodness-of-fit test (HL test). A decision curve analysis (DCA) was performed, and the nomogram was compared with scoring systems commonly used during clinical practice to assess the net clinical benefit.

**Results:** Indicators included in the nomogram were age, OASIS score, SAPS II score, respiratory rate, partial thromboplastin time (PTT), cardiac arrhythmias, and fluid-electrolyte disorders. The results showed that our model yielded satisfied diagnostic performance with an AUC value of 0.902 and 0.883 using the training set and on internal validation. The calibration curve and the Hosmer-Lemeshow goodness-of-fit (HL). The HL tests exhibited satisfactory concordance between predicted and actual outcomes (*P* = 0.648). The DCA showed a superior net clinical benefit of our model over previously reported scoring systems.

**Conclusion:** In summary, we explored the incidence of mortality during the ICU stay of thoracic fracture patients without neurological compromise and developed a prediction model that facilitates clinical decision making. However, external validation will be needed in the future.

## Background

A spinal fracture is a dislocation or fracture of the vertebrae with an annual incidence of 26 per 100 000 (1), accounting for ~23.3% of all trauma patients (2). The most prevalent spinal fractures occur at the level of the lumber spine, followed by the thoracic spine (3). Motor vehicle accidents (MVC) and falls from heights are the most common causes of spinal fracture; the annual incidence of spinal fracture has increased, with the reported increase in motor vehicle crashes (4). In China, the incidence rate of spinal fractures in 2007 was twice that in 2001 (3).

Numerous studies have investigated the long-term prognosis of spinal fractures using data from large patient populations in recent years. In patients with thoracolumbar fractures exhibiting mild symptoms, the visual analog scale (VAS) scores and the Roland Morris Disability Questionnaire demonstrated poor results and a poor outcome in 6% of patients 10 years after non-operative treatment (5). Furthermore, for patients exhibiting more severe symptoms requiring surgery, only 50% reportedly return to their original jobs postoperatively (6).

Meanwhile, long-term mortality associated with spinal fractures is significantly increased in post-traumatic patients compared to those with no history of trauma (7), especially in osteoporotic patients (8, 9). It has also been shown that old age and male gender increase the long-term mortality risk after thoracolumbar fracture (10). Interestingly, studies have shown that trauma patients in the ICU have high mortality rates, as high as 31% (11). Notwithstanding that substantial progress has been made in trauma advanced life support over the years, the mortality rates are still high (12). Patients with concomitant spinal cord injury are more likely than those without neurological damage to present with multiorgan injury and die from secondary infection (13). Most importantly, many patients experience death prior to hospitalization due to severe spinal cord injuries (14). To the best of our knowledge, the prognosis of acute severe spine fracture patients in ICU has been largely unexplored, and current research hot spots still focus on osteoporosis, bone cement, biomechanical analysis, and so on. Meanwhile, the interest of intensivists in trauma patients has mostly focused on rib fractures and pelvic fractures (15–17). Due to the lack of adequate attention to such patients, a large number of high-risk patients are not identified in time for admission, which often leads to poor prognosis and even higher mortality. Little is currently known about the risk factors of poor prognosis in ICU patients with thoracic spine fractures, which has resulted in high mortality rates. Accordingly, we selected this specific patient population for our study.

It is essential to develop a prediction model based on routine clinical and laboratory parameters to ensure that it can be easily implemented during clinical practice. It has been established that nomograms can provide evidence-based and personalized risk estimates and contribute to clinical management and prognosis evaluation (18–21). This study aimed to develop a prediction model to predict mortality during the ICU stay of thoracic fracture patients without neurological compromise based on the Medical Information Mart for Intensive Care III (MIMIC-III) (22) clinical database.

## Materials and Methods

### Data Source

Our data were derived from the MIMIC-III database, a database established and open source by the Massachusetts Institute of Technology (MA, USA) containing information on more than 58,000 patients that attended the Beth Israel Deaconess Medical Center. We completed a web course offered by the National Institutes of Health (NIH) and were granted access to the MIMIC-III database (certification number: 42442549). The data were extracted from the MIMIC-III database using structure query language (SQL) with pgAdmin4 PostgreSQL 9.6.

### Study Population

**Figure 1:**
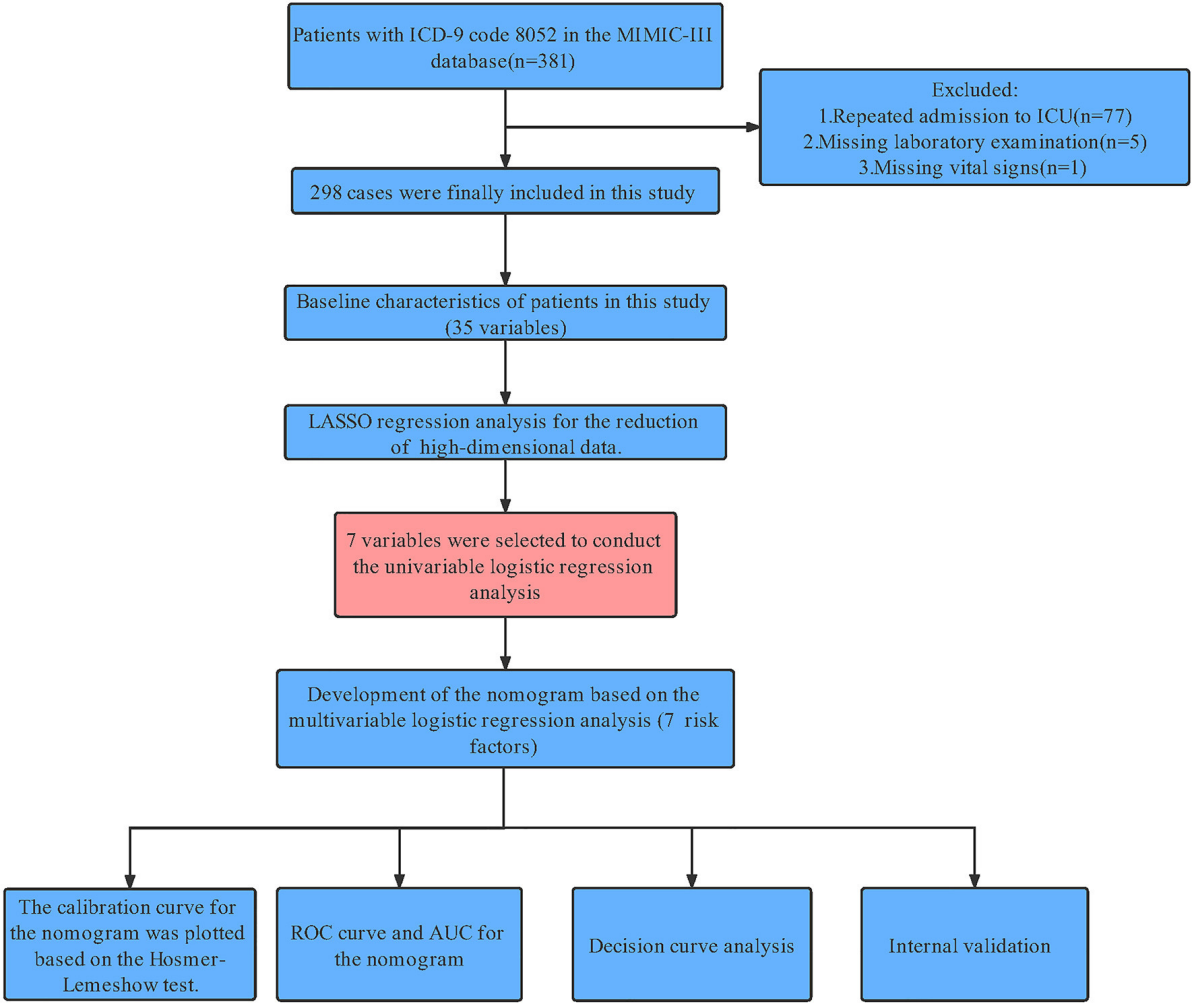
Workflow of the study. MIMIC-III, Medical Information Mart for Intensive Care III; ICU, intensive care unit; LASSO, least absolute shrinkage and selection operator; AUC, area under the curve; HL test, Hosmer-Lemeshow goodness-of-fit test; DCA, decision curve analysis.

Structured Query Language (SQL) with PostgreSQL (version 9.6 University of California, Berkeley) was used to extract information associated with each patient's unique HADM_ID from the MIMIC-III database. Through International Classification of Diseases 9th Edition (ICD-9) code = 8,052, we obtaining 381 patient. For patients with multiple admissions, we retained information only on the patient's first admission to the ICU. Patients with missing data >20% for laboratory tests (*n* = 5) and ital signs (*n* = 1) were excluded. Finally, 298 patients were included in the study (Figure 1).

### Clinical Variables and Definition

Using the patient's HADM_ID and ICUSTAY_ID as described above, we extracted the following data: demographics, vital signs, laboratory tests, comorbidities, and scoring systems. Among these, demographics included age and gender; vital signs included heart rate (HR), systolic blood pressure (SBP), diastolic blood pressure (DBP), mean blood pressure (MBP), respiratory rate (RR), temperature, SpO_2_, bicarbonate; laboratory tests included blood urea nitrogen (BUN), chloride, creatinine, glucose, hemoglobin, potassium, platelet, partial thromboplastin time (PTT), prothrombin time (PT), sodium, white blood cell (WBC), red blood cell (RBC); comorbidities included congestive heart failure, cardiac arrhythmias, liver disease, coagulopathy, fluid-electrolyte disorders, hypertension, renal failure, obesity, chronic pulmonary; scoring systems include scoring systems included simplified acute physiology score II (SAPS II), sequential organ failure as severity (sofa) score, oxford acute severity of illness score (OASIS), and the Glasgow coma scale (GCS). Indicators with >20% missing data were removed, such as height, weight, calcium; A deletion was also made for some complications that were not present in all patients, such as peptic ulcer, lymphoma, metastatic cancer. All the variables mentioned above were collected within 24 h of patients' admission.

### Statistical Analysis

We used the median and interquartile range to express continuous variables, while Wilcoxon's rank-sum test was selected for comparison between two groups; The categorical variables were expressed as frequency and proportion. Chi-square tests or Fisher's exact test was chosen for inter-group comparison according to the situation. We selected the bootstrap method for internal validation based on the number of patients, in accordance with the transparent reporting of a multivariable prediction model for individual prognosis or diagnosis (TRIPOD) guidelines (23). We used the neighbor interpolation method in the MICE R package (24) to fill in missing data. Then, the least absolute shrinkage and selection operator (LASSO) expression was used for screening predictors of mortality (25). For the cross-validation results, we selected lambda = min to determine the final candidate variables (26, 27). The multivariate logistic regression model was established using these variables, and multicollinearity was evaluated by variable inflation factors (VIF). The area under the receiver operating curve (AUC), sensitivity and specificity were used to evaluate the model's performance. The Youden index determined the best cutoff point. Finally, the nomogram was plotted using the R package “regplot.” The calibration C index (bootstrap resampling 1,000 times) (28), the calibration curve (relationship between observation probability and prediction probability), Hosmer-Lemeshow goodness of fit test (HL test), and brier score were used to evaluate the degree of consistency between observed and predicted outcomes. Decision curve analysis (DCA) was used to assess the net clinical benefit (29). All statistical analyses were completed using R language (version 3.6.3); a *p* < 0.05 was statistically significant.

## Results

### Patient Characteristics

Two hundred and ninety eight patients were finally included in the study, with an average age of 53.5 years. 34.6% (*n* = 103) were female, with a mortality rate of 13.6% while male patients accounted for 65.4% (*n* = 195), with a mortality rate of 7.2%. Table 1 compares the differences in characteristics between the in-hospital death group and the survival group. Compared with the survival group, patients in the death group were older, had lower body temperature and blood pressure, faster respiratory rate, and lower bicarbonate. Meanwhile, lower hemoglobin and platelet counts were observed in the death group, while PT and PTT values were relatively higher, indicating poor coagulation function. The OASIS, SOFA, and SAPS II scores were significantly higher, with a higher prevalence of cardiac arrhythmias, coagulopathy, and fluid-electrolyte disorders.

**Table 1:** Baseline characteristics of in-hospital death and survival groups.

**Variable**	**Total (***n*** = 298)**	**Survival (***n*** = 270)**	**Death (***n*** = 28)**	***P*** **-value**
Age, years	53.50 [35.00, 72.00]	50.00 [32.25, 68.00]	76.50 [66.25, 80.00]	<0.001
Female, *n* (%)	103 (34.6)	89 (33.0)	14 (50.0)	0.111
HR, beats/min	146.50 [74.25, 219.75]	145.50 [75.25, 220.75]	149.00 [71.50, 200.75]	0.744
SBP, mmHg	120.22 [111.13, 133.15]	121.19 [111.81, 134.21]	109.43 [102.06, 118.81]	<0.001
DBP, mmHg	63.33 [57.08, 70.51]	64.40 [57.75, 70.83]	58.86 [53.31, 63.82]	0.019
MBP, mmHg	80.25 [73.24, 87.63]	81.04 [74.10, 88.55]	74.01 [70.58, 77.88]	0.001
RR, breaths/min	17.84 [15.88, 20.95]	17.65 [15.79, 20.63]	20.75 [17.42, 23.28]	0.001
Temperature, °C	37.00 [36.56, 37.41]	37.05 [36.60, 37.43]	36.66 [36.16, 37.36]	0.041
PO2, mmHg	97.85 [96.41, 99.05]	97.87 [96.41, 99.07]	97.54 [96.65, 98.99]	0.714
Bicarbonate, mmol/L	23.00 [21.00, 26.00]	24.00 [21.00, 26.00]	21.00 [17.75, 24.00]	0.007
BUN, mg/dl	15.00 [12.00, 20.00]	15.00 [12.00, 20.00]	16.00 [14.00, 24.25]	0.174
Chloride, mEq/L	107.00 [103.00, 109.75]	106.00 [103.00, 109.00]	108.50 [101.00, 112.75]	0.129
Creatinine, mg/dL	0.90 [0.70, 1.10]	0.90 [0.70, 1.10]	0.90 [0.70, 1.20]	0.955
Blood glucose, mEq/L	136.00 [113.00, 161.00]	134.00 [112.00, 160.75]	147.50 [133.00, 172.50]	0.066
Hemoglobin, g/dl	12.35 [11.00, 13.78]	12.40 [11.12, 13.80]	10.90 [9.17, 13.33]	0.01
Platelet, K/μL	221.50 [175.00, 282.75]	224.00 [182.00, 288.00]	178.50 [135.75, 239.25]	0.003
Potassium, mEq/L	4.10 [3.80, 4.50]	4.10 [3.73, 4.40]	4.15 [3.80, 4.90]	0.519
PT, s	13.30 [12.50, 14.50]	13.30 [12.50, 14.38]	15.05 [13.18, 18.05]	0.002
PTT, s	26.30 [23.83, 29.80]	26.05 [23.72, 29.20]	33.50 [27.42, 43.73]	<0.001
RBC, m/μL	4.00 [3.59, 4.47]	4.02 [3.63, 4.49]	3.75 [2.92, 4.26]	0.005
Sodium, mEq/L	140.00 [138.00, 142.00]	140.00 [138.00, 141.00]	141.00 [136.00, 142.25]	0.413
WBC, K/μL	14.35 [10.50, 18.30]	14.25 [10.50, 18.25]	15.05 [11.45, 19.15]	0.541
Congestive heart failure, *n* (%)	20 (6.7)	18 (6.7)	2 (7.1)	1
Cardiac arrhythmias, *n* (%)	43 (14.4)	33 (12.2)	10 (35.7)	0.002
Hypertension, *n* (%)	8 (2.7)	7 (2.6)	1 (3.6)	1
Chronic pulmonary, *n* (%)	34 (11.4)	30 (11.1)	4 (14.3)	0.849
Renal failure, *n* (%)	10 (3.4)	9 (3.3)	1 (3.6)	1
Liver disease, *n* (%)	7 (2.3)	6 (2.2)	1 (3.6)	1
Coagulopathy, *n* (%)	14 (4.7)	10 (3.7)	4 (14.3)	0.04
Obesity, *n* (%)	11 (3.7)	9 (3.3)	2 (7.1)	0.623
Fluid-electrolyte disorders, *n* (%)	61 (20.5)	48 (17.8)	13 (46.4)	0.001
OASIS	32.00 [26.00, 38.00]	31.00 [25.00, 37.00]	41.00 [36.75, 47.25]	<0.001
GCS	15.00 [14.00, 15.00]	15.00 [14.00, 15.00]	15.00 [14.00, 15.00]	0.331
SOFA	3.00 [1.00, 5.00]	2.00 [1.00, 4.00]	6.00 [3.75, 8.25]	<0.001
SAPSII	28.00 [19.00, 37.00]	26.50 [18.00, 35.00]	46.00 [37.75, 52.25]	<0.001

*HR, heart rate; SBP, systolic blood pressure; DBP, diastolic blood pressure; MBP, mean blood pressure; RR, respiratory rate; BUN, blood urea nitrogen; PT, prothrombin time; PTT, partial thromboplastin time; RBC, red blood cell; WBC, white blood cell; SAPSII, simplified acute physiology score II; SOFA, sequential organ failure; OASIS, oxford acute severity of illness score; GCS, Glasgow coma scale*.

### Characteristics Selection and Development of a Nomogram

**Figure 2:**
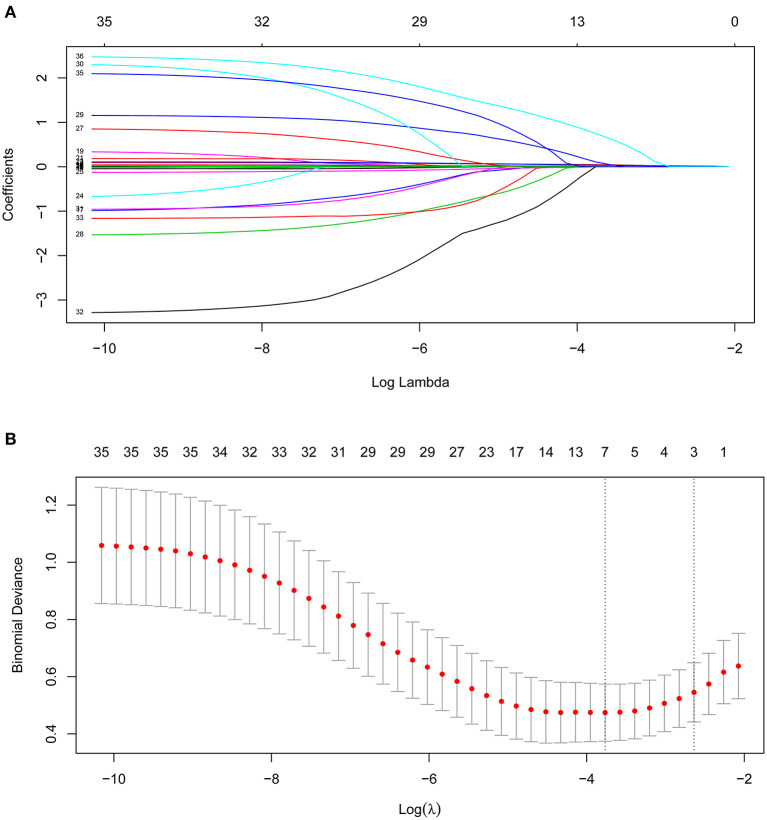
Clinical variables were selected using the lasso logistic regression model. **(A)** Tuning parameter (λ) selection using LASSO penalized logistic regression with 10-fold cross-validation. **(B)** LASSO coefficient profiles of the radiomic features.

**Figure 3:**
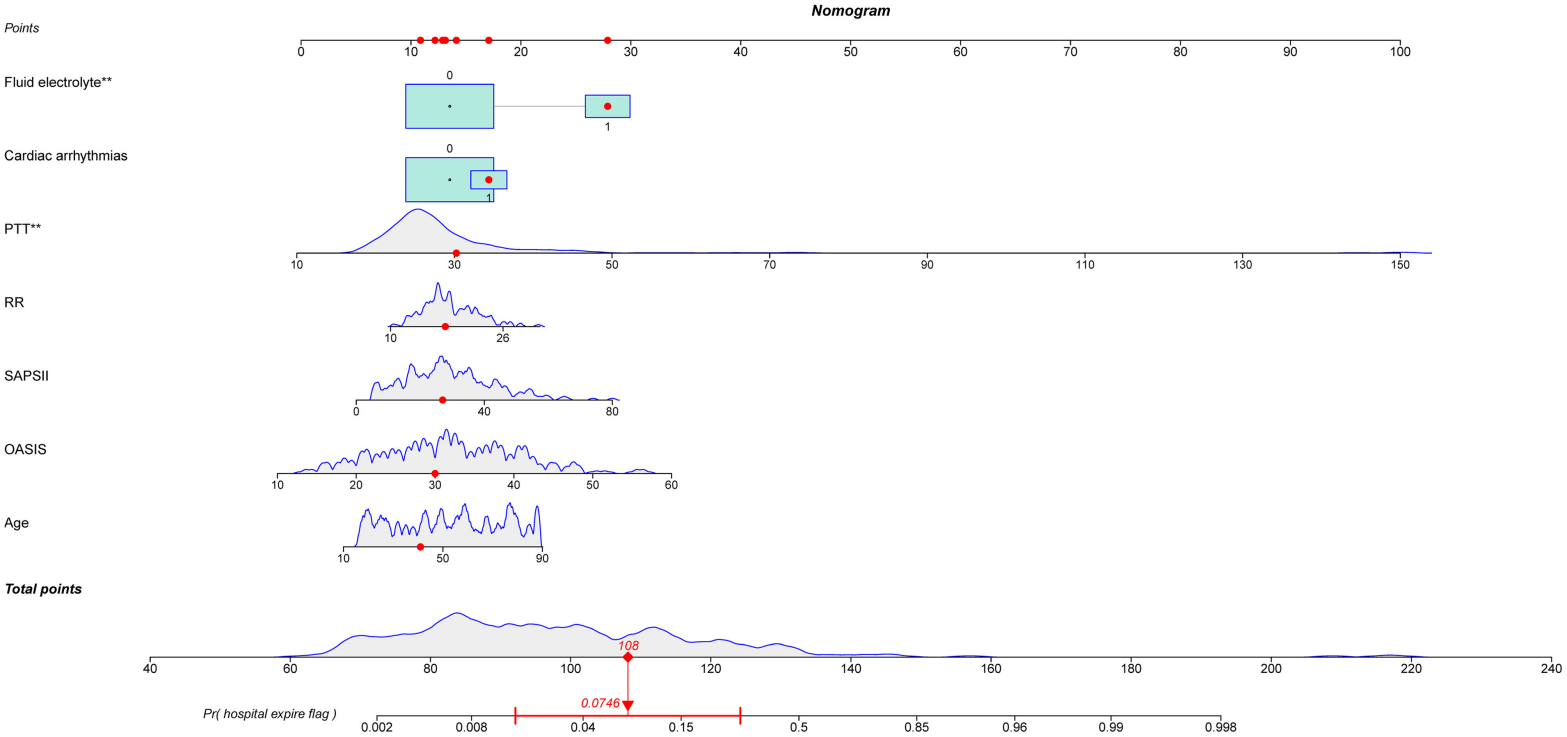
Nomogram to predict the risk of in-hospital mortality in patients with thoracic spine fractures without neurological injury in the ICU. PTT, partial thromboplastin time; RR, respiratory rate; SAPS II, simplified acute physiology score II; OASIS, oxford acute severity of illness score. ** means *p* < 0.01.

Out of 35 variables, seven remained in the lasso logistic regression model based on the binomial deviance minimum criteria (ratio 5:1) (Figure 2). The final seven variables included in the multivariate logistic regression, were: age (OR: 1.02; 95% CI 0.99–1.06), OASIS score (OR: 1.08; 95%CI 1.00–1.17), SAPS II score (OR: 1.03; 95% CI 0.98–1.08), RR (OR: 1.07; 95% CI 0.94–1.20), PTT (OR: 1.08; 95%CI 1.03–1.13), cardiac arrhythmia (OR: 1.44; 95%CI 0.43–4.62), and fluid-electrolyte disorders (OR: 4.40; 95% CI 1.62–12.54) (Table 2). Based on this model, we constructed a nomogram to predict mortality in ICU patients with thoracic spine fractures without neurological injuries (Figure 3).

**Table 2:** Multivariate regression model based on LASSO regression results.

**Variables**	**Multivariable logistics model**		
	**Coefficients**	**OR (95%CI)**	***P*** **-value**
Age	0.02334	1.02 (0.99–1.06)	0.1627
OASIS	0.074	1.08 (1.00–1.17)	0.069
SAPSII	0.03004	1.03 (0.98–1.08)	0.2284
RR	0.06601	1.07 (0.94–1.20)	0.288
PTT	0.07398	1.08 (1.03–1.13)	0.0028
Cardiac arrhythmias	0.36681	1.44 (0.43–4.62)	0.5394
Fluid-electrolyte disorders	1.48207	4.40 (1.62–12.54)	0.0041

*OASIS, oxford acute severity of illness score; SAPSII, simplified acute physiology score II; RR, respiratory rate; PTT, partial thromboplastin time*.

### Apparent Performance of the Nomogram and Web Calculator

**Figure 4:**
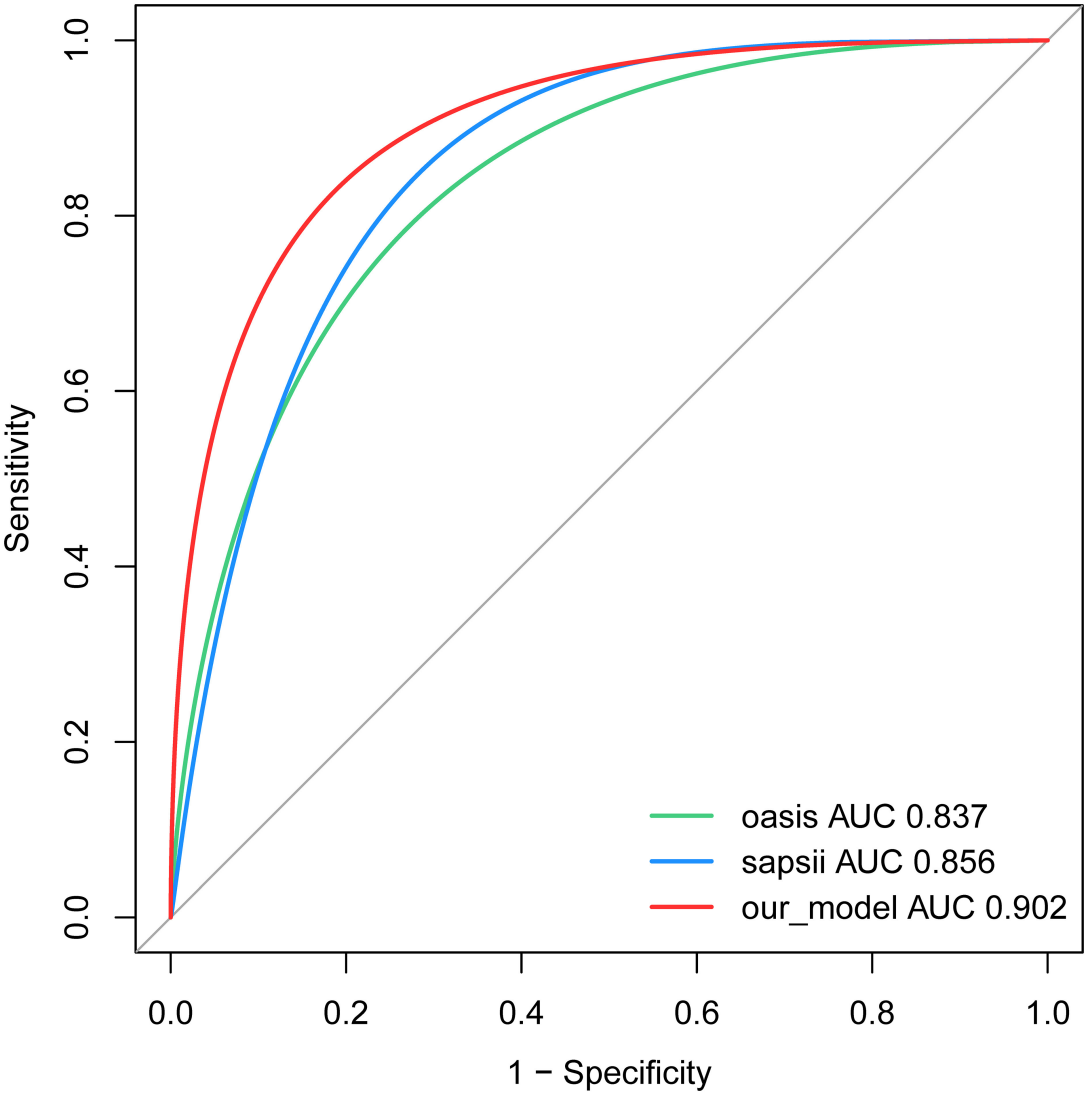
Receiver operating characteristic curve of the nomogram. AUC, area under curve; OASIS, oxford acute severity of illness score; SAPS II, simplified acute physiology score II.

**Figure 5:**
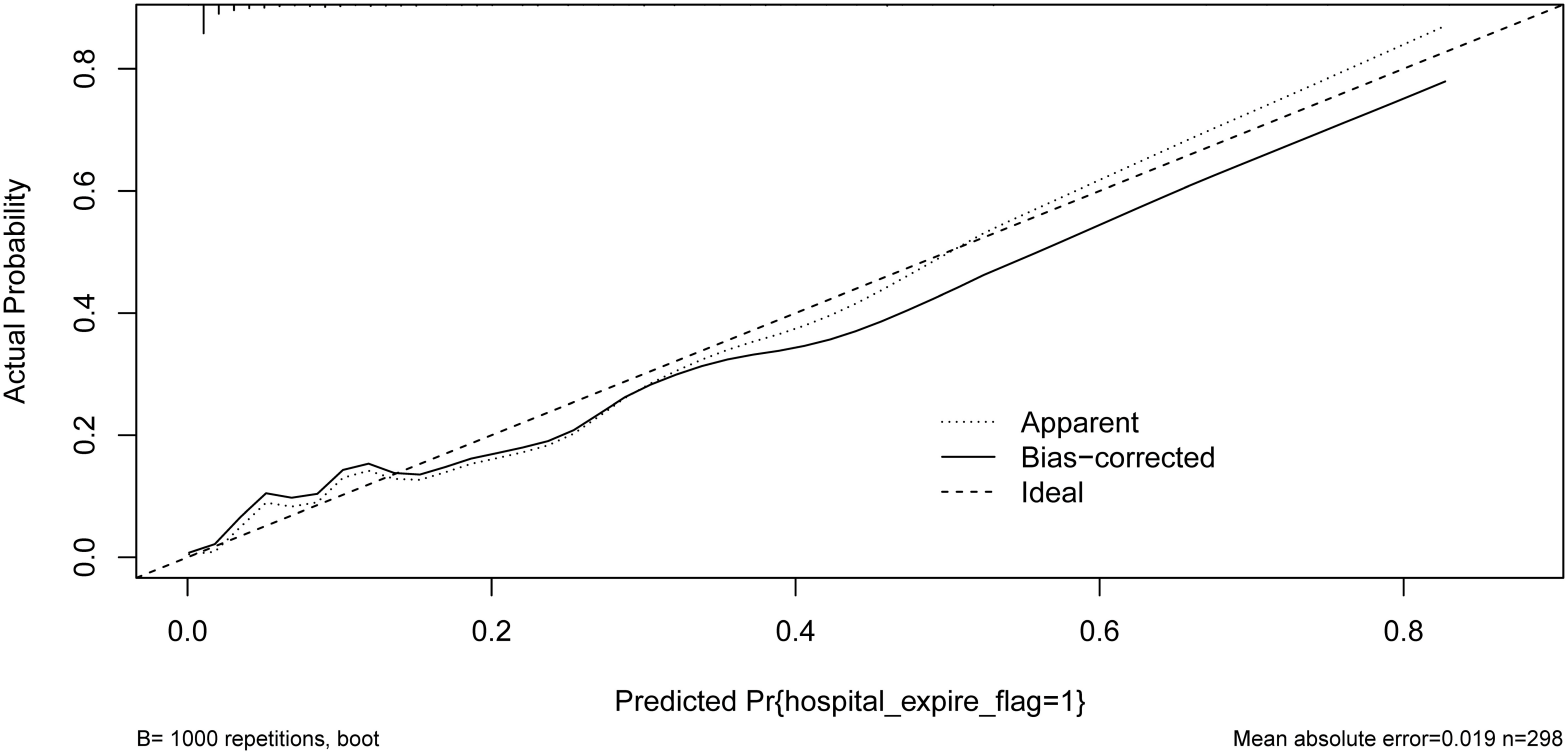
Calibration curves of the predicted nomogram. The dashed line represents the original performance, and the solid dashed line represents the performance during internal validation by bootstrapping (*B* = 1,000 repetitions). Results of the Hosmer-Lemeshow test demonstrate that the *P*-value was 0.648.

Our model yielded an AUC value of 0.902 (95% CI 0.849–0.959), with a C-index of 0.883 after 1,000 bootstrap resampling internal validations. According to the Youden index, the optimal cutoff value was 18.45%, with a sensitivity and specificity of 0.870 and 0.786, respectively (Figure 4). Meanwhile, the calibration curve showed a good fit during internal validation (Figure 5), while the HL test showed that our predicted and observed values were close (*P* = 0.648); The Brier score was 0.0543 and 0.0623 after bootstrap correction. The ROC values of the scoring systems SAPS II and OASIS incorporated into the regression were 0.856 and 0.837, respectively, suggesting that our model exhibited better predictive performance than scoring systems commonly used clinically. To facilitate clinical use, we constructed a web calculator (https://ouyyjlueducn.shinyapps.io/dynnomapp/) based on the model.

### Clinical Practice

**Figure 6:**
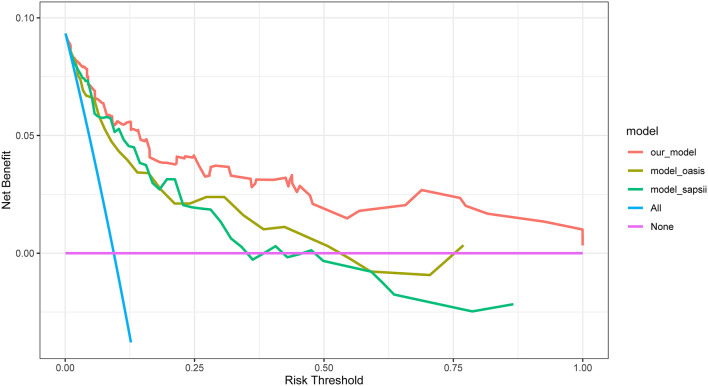
DCA for our model, OASIS and SAPS II. The y-axis measures the net benefit. DCA, decision curve analysis; OASIS, oxford acute severity of illness score; SAPS II, simplified acute physiology score II.

DCA of the nomogram was performed (Figure 6). The blue curve in the figure represents that all the patients received intervention, the straight purple line represents that none patients receive the intervention, and the red curve represents the clinical benefit of our model. For our model, when the predicted probability threshold was set to 0.065%, the net clinical benefit was 9.39%. Our results showed that our model had a superior net clinical benefit than the OASIS and SAPS II scoring systems.

## Discussion

Fractures have become a major public health problem in most countries (30), especially spine fractures, which account for approximately 46% of spinal injuries in severe trauma (31, 32). In this regard, patients that sustain spine fractures have the poorest functional prognosis and potential to return to work (33). Although many studies have discussed the long-term prognosis of spinal fractures, to the best of our knowledge, this is the first study to investigate the mortality risk of spinal fracture patients without neurological impairment. The injury severity score (ISS) has previously been used to guide the risk stratification of trauma patients; however, some studies have pointed out that this scoring system was not reliable (34) since it did not consider the characteristics of patients with spinal trauma. We summarized the related works in Table 3. Previous methods of classifying spinal trauma have been most relevant in guiding surgical treatment (35), and commonly include the Trauma and Injury Severity Score (ISS) and the New Trauma and Injury Severity Score (NISS). However, none of these assessment methods take into account the individual spinal trauma patient, and our work fills a gap in the risk stratification of patients with severe spinal trauma. After internal validation of the model, we found that the predictions of the model were in good agreement with the actual results. More interestingly, the constructed model had better discrimination and net clinical benefit compared to the previously used OASIS and SAPSII systems. Also, based on the reviewers' comments, we have done more work. To facilitate clinical use, we constructed a web calculator (https://ouyyjlueducn.shinyapps.io/dynnomapp/) based on the model. In summary, we provide an easy-to-use model for this group of patients, which can identify high-risk patients early, take appropriate interventions early, and reduce poor prognosis and in-hospital mortality.

**Table 3:** The summary of the previous related work.

**ID**	**Title**	**Author**	**Journal**	**Public date**
1	Thoracic spine fracture in the panscan era	Remy Bizimungu et al.	Ann Emerg Med	2020 Aug
2	Early risk stratification of in hospital mortality following a ground level fall in geriatric patients with normal physiological parameters	Nasim Ahmed et al.	Am J Emerg Med	2020 Dec
3	Mortality and cause of death in patients with vertebral fractures: a longitudinal follow-up study using a national sample cohort	Hyo Geun Choi et al.	Spine (Phila Pa 1976)	2020 Mar
4	Predicting survival in older patients treated for cervical spine fractures: development of a clinical survival score	Darryl Lau et al.	Spine J	2019 Mar
5	Long-term post-traumatic survival of spinal fracture patients in northern Finland	Ville Niemi-Nikkola et al.	Spine (Phila Pa 1976)	2018 Dec
6	Spinal fractures in older adult patients admitted after low-level falls: 10-year incidence and outcomes	Randeep S. Jawa et al.	J Am Geriatr Soc	2017 May
7	Height loss in older women: risk of hip fracture and mortality independent of vertebral fractures	Teresa A. Hillier et al.	J Bone Miner Res	2012 Jan
8	Mortality and incident vertebral fractures after 3 years of follow-up among geriatric patients	H. C. van der Jagt-Willems et al.	Osteoporos Int	2013 May
9	Predicting in-hospital mortality in elderly patients with cervical spine fractures: a comparison of the Charlson and Elixhauser comorbidity measures	Mariano E. Menendez et al.	Spine (Phila Pa 1976)	2015 Jun
10	Characteristics and outcomes of hospitalized patients with vertebral fragility fractures: a systematic review	Terence Ong et al.	Age Aging	2018 Jan
11	Mortality after vertebral fractures in a Japanese population	Yuzo Ikeda et al.	J Orthop Surg (Hong Kong)	2010 Aug
12	Is radiographic vertebral fracture a risk factor for mortality?	Daniel W. Trone et al.	Am J Epidemiol	2007 Nov
13	Long-term morbidity and mortality after a clinically diagnosed vertebral fracture in the elderly–a 12- and 22-year follow-up of 257 patients	R. Hasserius et al.	Calcif Tissue Int	2005 Apr
14	Incidence of acute care complications in vertebral column fracture patients with and without spinal cord injury	D. J. Fletcher et al.	Spine (Phila Pa 1976)	1995 May

With the rapid development of computer technology in recent years, the combination of machine learning technology and medical practice has become a major trend (19–21, 26, 27). Along with the continuous innovation of convolutional algorithms, from LeNet by Lecun et al. (36) to ResNet by He et al. (37), computer-aided decision making through imaging has become a hot topic in medical research, such as prediction of BMI by facial image features to predict BMI (38) and fundus images to predict diabetic retinopathy (39). In addition, the prevalence of electronic medical records and the establishment of large medical databases have also provided the basis for research on clinical problems, and the combination with machine learning has shown remarkable performance in predicting the occurrence and prognosis of diseases (40, 41). Unfortunately, however, there is still a lack of research in the current field for our patient population. It is therefore necessary to develop an easy-to-use and reliable tool to inform clinical practice. In the present study, we established a nomogram consisting of seven predictors: age, OASIS score, SAPS II score, RR, PTT, cardiac arrhythmias, fluid-electrolyte disorders. Multiple indicators used during the validation process, including AUC, calibration curve, HL test and DCA, indicated that our nomogram yielded satisfactory prediction results.

As seen in Table 1, significant differences in coagulation markers PT, PTT, and coagulopathy were found between the death and the survival groups, especially PTT, which was an important predictor of mortality. Trauma-induced coagulopathy (TIC) has been reported to play an important role in trauma healing (42), and an increasing body of evidence suggests that uncontrolled hemorrhage is a preventable cause of death in the early stages after injury (43–45), which has been reported to be as high as 40–80% (46). The incidence rate of TIC often correlates with the severity of tissue injury (47) since tissue injury releases a large number of damage-associated molecular patterns (DAMPs), which promote multiple inflammatory pathways and thereby affect coagulation (42). Endothelial dysfunction has been reported to participate in TIC (48), and plasma samples from severely injured patients on admission exhibited increased levels of syndecan-1, which correlated with increased activated protein C (APC), prolonged PTT, and elevated epinephrine levels (49). Furthermore, platelet defects and dysfunction are also important contributors to TIC (50). In our study, even though the platelet count in the deceased group was still within the normal range, a significant decrease was observed (Table 2), suggesting an association between a relatively low platelet count and increased mortality, consistent with previous studies (51, 52). Similarly, a prospective study demonstrated that 91% of patients with severe injuries had platelet dysfunction despite normal platelet counts (53).

The most common arrhythmia in patients with chest trauma or surgery is atrial fibrillation, often associated with longer ICU stay and higher mortality (54). On the one hand, most patients with pre-existing atrial fibrillation receive anticoagulant therapy. When combined with previously described coagulation dysfunction, fatal bleeding can result from small traumas (55). On the other hand, patients with new-onset atrial fibrillation have higher in-hospital mortality than patients with previous atrial fibrillation (56) reportedly. The mortality among patients with arrhythmia in our study group was 23.3% (*n* = 10/43), much higher than that of patients without arrhythmia. Moreover, a significant difference in the number of patients with arrhythmia was found between the survival and death groups (*P* < 0.01).

It is widely acknowledged that the onset of arrhythmias is highly correlated with electrolyte disturbances (54). Many studies have demonstrated that electrolyte disturbances and vertebral fractures are associated with higher mortality, while elderly patients with fractures are more likely to have hyponatremia (57–59). Interestingly, in our study, albeit patients in the deceased group were much older than those in the survival group, no significant difference in sodium levels was found between the two groups. However, our study also confirmed higher mortality in patients who develop fluid-electrolyte disorders, emphasizing the need for early and effective fluid management in this subset of patients (60).

An analysis of nationwide patients with vertebral fractures in Japan revealed that advanced age is a significant risk factor for complications (OR 1.38) (61). We consistently found that advanced age patients were more likely to experience in-hospital death, which may be associated with sarcopenia (62), poor nutritional status (63), and development of fluid-electrolyte disorders, as mentioned earlier. However, we found that gender was not a relevant factor for in-hospital death in our study, which was inconsistent with the literature. This discrepancy could be accounted for by the fact that the subjects of our research were ICU patients, which were critically ill, while gender had more influence in long-term prognosis (10).

Due to its simplicity and ease of observation, the respiratory rate is one of the indicators traditionally used for the early identification of high-risk patients after trauma (64). A respiratory rate >20 was an important indicator in new evaluation criteria for trauma patients (65). Consistently, we found that tachypneic patients with a respiratory rate within 24 h of admission are more likely to experience in-hospital mortality and require early intervention. Two severity scoring systems, SAPS II and OASIS were also included in the prediction model of this study. At present, much controversy surrounds the predictive accuracy of these two scoring systems in orthopedic trauma patients (66, 67). In our present study, both scoring systems were integrated into our nomogram. The ROC plots showed that our model outperformed these two scoring systems in discrimination (Figure 3). At the same time, DCA exhibited greater net clinical benefit than these two scoring systems (Figure 5).

There are still limitations that need to be considered. First, these data were from a public database spanning 2007–2014. Therefore, the model needs external validation from different medical Institutions. Second, because missing data is >20% in the dataset, there is a lack of some important clinical parameters. Finally, although nomogram is already widely used in clinical practice to aid medical decision making, we want to further simplify the work and expand the scenarios in which it can be used. Therefore, in the future we hope to package predictive tools into applications for mobile devices, wearables, or personal computers.

## Conclusion

Our study found that age, OASIS score, SAPS II score, RR, PTT, cardiac arrhythmias, and fluid-electrolyte disorders are predictors of mortality during the ICU stay of thoracic fracture patients without neurological compromise. A multiple logistic regression model and a nomogram were developed and validated. During clinical practice, this nomogram could help physicians screen high-risk patients, make optimal use of resources, and decrease the occurrence of death in this patient population.

## Data Availability Statement

The raw data supporting the conclusions of this article will be made available by the authors, without undue reservation.

## Author Contributions

HW collected the data, analyzed the data, and drafted the manuscript. TF, YO, MK, JZ, and RD conceived of the study, participated in its design and coordination, and helped to draft the manuscript. YQ was responsible for the whole project, reviewed the manuscript, designed the study, and supervised the study. All authors contributed to the article and approved the submitted version.

## Funding

This work was supported by Norman Bethune Program of Jilin University (JJKH20201101KJ).

## Conflict of Interest

The authors declare that the research was conducted in the absence of any commercial or financial relationships that could be construed as a potential conflict of interest.

## Publisher's Note

All claims expressed in this article are solely those of the authors and do not necessarily represent those of their affiliated organizations, or those of the publisher, the editors and the reviewers. Any product that may be evaluated in this article, or claim that may be made by its manufacturer, is not guaranteed or endorsed by the publisher.
